# The renal hydatid cyst: report on 4 cases

**DOI:** 10.4314/pamj.v8i1.71147

**Published:** 2011-03-22

**Authors:** Mohamed Rami, khalid Khattala, Aziz ElMadi, My Abderrahmane Afifi, Youssef Bouabddallah

**Affiliations:** 1 Department of Pediatric Surgery, CHU Hassan II, Fes, Morroco

**Keywords:** Hydatid cyst, kidney, child, Morocco

## Abstract

The hydatid cyst is a frequent pathology in Morocco. All localizations are possible. However, renal hydatid cyst is still rare; it constitutes about 2.5 % of all localizations. We report 4 cases admitted at the Department of Pediatric Surgery of the University Hospital of Fes in the period running from February 2004 to January 2008. The four patients were of ages ranging from 8 to 11 years old. Two of them had double localization in the kidney and liver. The imaging was the diagnostic tool of choice. The patients benefited from surgical treatment; two were treated using standard surgery while laparoscopic surgery was used in the next two. Anti-parasitic medication was associated in the post-surgery stage for a period of six months. The evolution was good for all the patients.

## Introduction

Echinococcosis or hydatid cyst disease of the kidney is extremely rare in children and constitutes only 2-4% of all cases of hydatid disease. Its diagnosis is easy and mainly based on ultrasound and CT-scan. The treatment is mainly surgical, by open surgery or by laparoscopic management. Here, we present 4 cases; 2, with only renal localization, were managed laparoscopically and the others by conventional surgery. The follow-up showed no residual disease.

## Patients and observations

**Observation 1**

**Figure 1:**
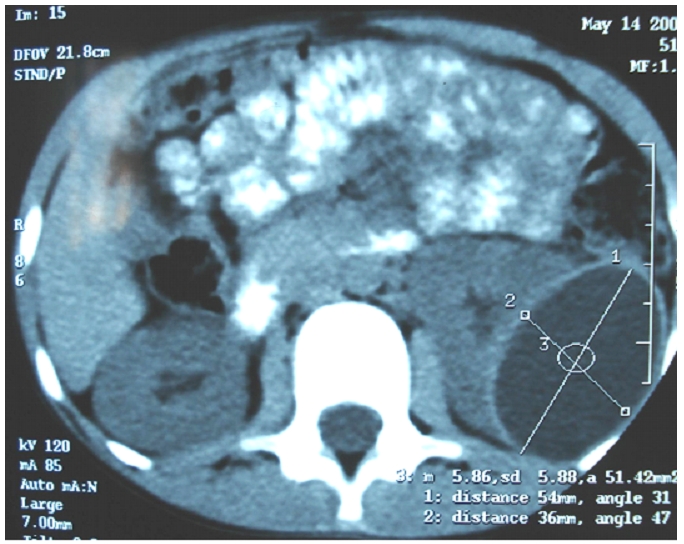
TDM aspect of a renal hydatid cyst

**Figure 2:**
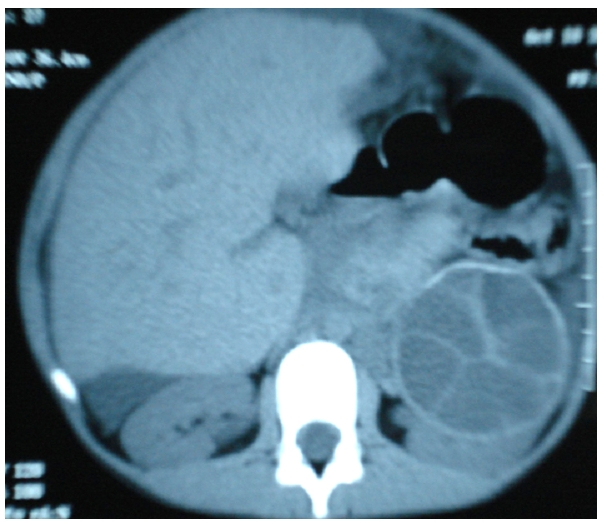
TDM aspect of a renal hydatid cyst after injection of contrast

An 8-year-old girl, without any noticeable medical history, was admitted into our hospital. The clinical profile consists haemoptysis sputum and without hydatid vomica or any other signs. She was admitted in pediatric department where she underwent phthisiologic investigations that were all negative. The biological assessment was normal. The clinical examination showed an apyretic patient with rattle at auscultation, the abdomen was supple without any hepatosplenomegaly or lumbar contact. The thoracic x-ray found a left basal site; hence the patient was initially treated using an antibiotherapy that improved the symptomatology. Abdominal ultrasound demonstrated a lowed polar renal cyst, while the abdominal CT-scan confirmed the hydatid character of the cyst (Figure 1 and 2). The patient was operated using celioscopy technique; after injection of 30% NaCl solution, the membrane encapsulating the germ was removed, a resection of the neighboring tissues was also realized, and closing after drainage. The evolution is good within a four years recession.

**Observation 2**

**Figure 3:**
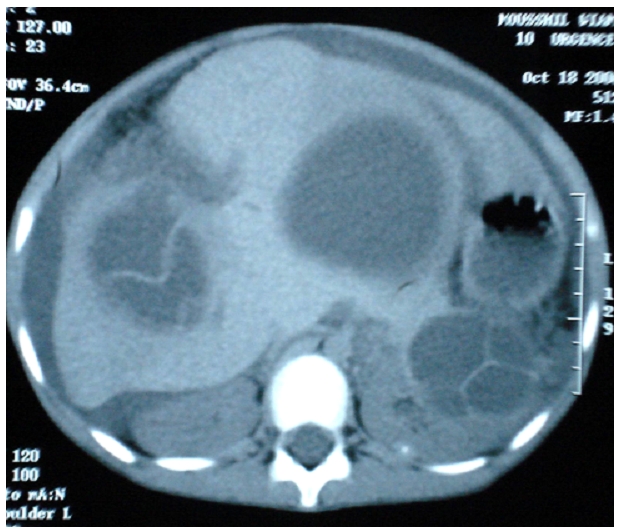
TDM showing hepatic and renal localization of hydatid cysts

A 10-year-old girl was admitted with recurring abdominal pains. The examination found a hepatomegaly with two entities of solid palpable masse without pain. A left lumbar sensitivity was also found without any urinary signs. The biological assessment was normal. The ultrasound found a hepatic hydatidosis (two cysts of 65× 47 mm and 70× 51 mm) and a renal localization (63× 51 mm) (Figure 3). The patient was operated for this double localization using a transversal umbilical incision. The patient has received albendazol-based medical treatment for six month. The evolution is simple within of three years recession.

**Observation 3**

An 11-year-old girl was treated for pulmonary tuberculosis when she was 3 years old; then operated on to remove a hepatic hydatid cyst while she was 8 years old. The patient was admitted at emergency department for an acute abdomen following an abdominal contusion. The examination found a febrile and vomiting patient with abdominal pain. The palpation objectified a painful hepatic mass with matity of the sides. The biological assessment found a hyperleukocytosis at 13000 elements/mm^3^. The ultrasound and the CT-scan of the abdomen revealed two hepatic hydatid cysts (80×70 mm and 66× 58 mm) including one that has already ruptured. A RHC of type 3 (67×54 mm) was also found in the left kidney.The patient was urgently operated on to remove the ruptured hepatic cyst. She developed a cutaneous rush with hypotension. The cure of the renal cyst was differed 2 months later to reduce the time of the intervention considering the anaphylactic shock developed, and to avoid sowing the retroperitoine. The patient favorably evolved under treatment, using corticosteroid and antiparasitic therapy, and she has received albendozale-based medical treatment for six months. The follow up is for three years.

**Observation 4**

A 10 year old boy was admitted for left lumbar pain and hematuria. The examination found a left lumbar mass. The ultrasound and CT-scan of the abdomen confirmed type 3 renal hydatid cyst. The patient was managed by cœlioscopy, after injection of 30% NaCl solution, the germinative membrane was removed, a resection of the neighboring tissues was also realized, the fluid content of the cyst was aspirated and the cavity was filled with 30% NaCl solution. The patient has received albendazol-based medical treatment for six months. The evolution is good within a follow up of one year.

## Discussion

The RHC is the third localization of the hydatid cyst after liver and lungs. The RHC represents about 2.5% of the whole localizations and most often unilateral and unique, however cases of multiple and even bilateral were reported [1,2]. Hydatid cyst might occur in disseminated form, such were two of the cases in our series [1-4]. The RHC might remain asymptomatic for years in case of slow evolvement, like in the first case [3-7]. Frequently, The RHC is revealed by an abdominal mass syndrome that is often associating signs varying from general to particular such as urinary, lumbar pain; dysury or hematuria [2,3,5]. The pathognomonic sign consisting of hydaturia should indicate the rupture of cyst and the diffusion of the content in the excretory tracts [4,8] we do not report this sign in our study.

The renal localization is discovered while investigating known hydatidosis extensions. In case of a large cyst, signs of compression of closer organs might appear, and would manifest in terms of dyspnea, abdominal swelling, dyspepsia and constipation. The radiological investigations allow clarifying the diagnosis and provide the most interesting evidences for hydatidosis diagnosis. The X-ray allows displaying of a thin arc-shaped calcification that is characteristic of hydatid cyst compared to heterogeneous and more or less diffused calcifications. The diagnosis of hydatid cyst using ultrasound is more reliable and it is specific up-to 80%. This technique provides an accurate size of the cyst, its topography, its structure, and diagnosing any associated abdominal lesions.

According to Gharbi and al, the hydatid cystic disease is classified in five distinguishable ultrasonographic types [11]. And the frequent aspect published in pediatric series is type I [11]. Despite its reliability, the ultrasonography has its own limitations since it might confound necrosed tumors with an altered hydatid cyst [10-12]. The CT-scan remains the most suitable examination. It could be ordered promptly, and mostly for differential diagnosis once the ultrasonographic IV and V types are declared. The CT-scan might find out the nature of the tumoral disorder, localizing the site and its correlation to the neighborhood tissue, the CT-scan would assess also the safe rate of the safe renal parenchyma for an eventual conservative surgery. The RHC is expressed as liquidated homogeneous or heterogeneous mass, the attenuation coefficient is not modified by the contrast agent injection, however an increased signal is observed in case of communication with excretory tracts in a slow mode [11,12]. The architecture of the RHC found in MRI and CT scan are similar [13].

The immunological study contributes to introduce the diagnosis; and it consists of immunoelectrophoresis, indirect immunofluorescence and ELISA. This assessment approach is reliable in more than 70% of cases [4,9]. The blood hypereosinophilia was assessed in more than 50% of cases, however this assessment is mostly lacking inconsistency and specificity [2,4,9].

In the epidemiological context, the radiological and biological data allow preparing the diagnosis in all cases. Nevertheless, the differential diagnosis is established with other origins of renal masses such simple cyst, calcified hematoma, cystic nephroblastoma and abscess of kidney [4,5,7].

Considering the insufficiencies of medical treatment used especially in cases of small cysts (lower than 30 mm), and the potential risk of interventional radiology, the treatment of RHC remains surgical. The choice of the treatment approach depends on three basic elements: the volume of the mass; the relation of this mass with neighboring tissues and the extra-renal and abdominal localization of another hydatid cyst. Therefore, the pure lombotomy is the most used approach, followed by the median means reserved to RHC associating extra-renal visceral localization. In the cases with double localization, we used the laparotomy.

At present, the coelioscopy is increasingly used as it provides the same outcome, less morbidity and a better esthetic result but only some studies were published. We use it in two cases, when the localization was only renal, using a retroperitoneal access with good results. Current literature indicates that laparoscopic surgery is safe, and gives further support to minimally invasive treatment of this condition, as reported in the literature in other organs. Once the cystic lesion is displayed, the sterilization has been achieved using parasiticidal such salted hypertonic serum at 30‰. The surgical treatment is achieved including an essential stage consisting of the cystectomy considering the ablation of the hydatid membrane and possibly small vesicles. The pericysticectomy is generally limited to the resection of the external casual part “the resection of the prominent dome”. A systematic shell does not allow opening the residual cavity and reduce the hemorrhagic risk [4,5]. The partial or total nephrectomy is exceptionally used in children. However, this approach is required in the cases found with almost completely destroyed kidney. Several papers have reported percutaneous drainage of RHC, however this approach still requires more recession, and this represent a drawback of this method [14]. Nevertheless, the prognosis remains still very good whenever other localizations are not associated.

## Conclusion

The renal hydatid cyst in children constitutes a very rare localization. It has variable and polymorph symptomatology whish is rarely specific. The RHC has to be evoked in all renal cystic lesions, especially in endemic regions of the earth. The surgical approach remains the treatment of choice; particularly using laparoscopy and the resection should be mostly conservative.

## Competing interests

The authors declare that they have no competing interests

## Authors’ contribution

All the authors contributed at the diagnosis of this disease, in the treatment, and the follow-up of the patients.
